# Potential therapeutic effect of pomegranate seed oil on ovarian ischemia/reperfusion injury in rats

**DOI:** 10.22038/ijbms.2018.30149.7268

**Published:** 2018-12

**Authors:** Muhammed Yayla, Damla Cetin, Yasemen Adali, Pinar Aksu Kilicle, Erdem Toktay

**Affiliations:** 1 Department of Pharmacology, Faculty of Medicine, Kafkas University, Kars, Turkey; 2 Department of Pathology, Highlited sentences should be cahanged as Canakkale Onsekiz Mart University Faculty of Medicine, 17100 Canakkale/Turkey; 3 Department of Biology, Canakkale Onsekiz Mart University Faculty of Medicine, 17100 Canakkale/Turkey; 4 Department of Histology and Embryology, Ataturk University, Faculty of Medicine, 25240 Erzurum/Turkey

**Keywords:** Ischemia/Reperfusion, Oxidative stress, Ovary, Pomegranate seed oil, Punicaceae, Rats

## Abstract

**Objective(s):**

The aim of this study is to determine the therapeutic effects of pomegranate seed oil, which is a powerful antioxidant and anti-inflammatory agent, on ovarian-ischemia and reperfusion injury in rats.

**Materials and Methods:**

Fifty-six female albino Wistar rats were divided into 7 equal groups. Group 1; Sham Operation, Group 2; Ischemia, Group 3; Ischemia + Reperfusion, Group 4; Ischemia + Pomegranate 0,32 ml / kg (IP), Group 5; Ischemia + Pomegranate 0.64 ml / kg, Group 6; Ischemia + Pomegranate 0,32 ml / kg + reperfusion, Group 7; Ischemia + Pomegranate 0,64 ml / kg + reperfusion. Three hours after ischemia and 3 hours after reperfusion, the study was terminated.

**Results:**

While NADPH oxidase activity, MDA and TNF-α levels were significantly increased, SOD activity and GSH levels were reduced in ischemia and I/R groups. Low dose pomegranate seed oil application reduced significantly oxidative stress and NADPH oxidase activity in both ischemic and ischemic/reperfusion groups. At the same time, low-dose pomegranate seed oil extract reduced TNF-α levels and significantly increased antioxidant activity.

**Conclusion:**

PSO demonstrated an important therapeutic effect in the treatment of ovarian ischemia and reperfusion injury.

## Introduction

Development of ischemia in the vessels supplying the ovary is serious health problems that cause especially young girls to be sterile and lead to psychosocial disorders in the world (1). Ovarian cysts, pregnancy, polycystic over syndrome and transient or permanent obstruction of the ovarian artery may cause ischemia. However, the most common pathologic condition that eventuate as ovarian ischemia is ovarian torsion (1). If timely and adequate surgical and medical intervention done, fertility can be maintained by avoiding tissue damage (2). During ovarian ischemia, the energy production ceases in the absence of oxygen and resulting a series reactions with oxidative stress (3). Increased oxidative stress cause tissue damage which can be end up with necrosis or apoptosis (4, 5).

In the treatment of ischemia, it is aimed to reperfusion which can be identified as providing blood supply again (3). However, studies showed that injury is exacerbated during reperfusion (3, 6) because of increasing free oxygen radicals, endothelial damage and inflammation (3, 6, 7). Experimental and clinical studies carried out to overcome this damage have shown that agents with antioxidant activity can prevent oxidative stress-induced damage (8). Although there are many antioxidant agents in the literature, there is no effective treatment on the ovarian ischemia and reperfusion injury. Therefore, the development and studying of antioxidant and anti-inflammatory substances is also important for the future science.

Pomegranate (*Punica granatum*), an important member of the Punicaceae family, is known a fruit with many features since ancient times (9). Pomegranate consists of 3 parts; seed (3% of the weight), water (30% of the weight) and peel (9). In recent years significant progress has been made in the identification of the chemical components of pomegranate and their pharmacological effects (10). Pomegranate seeds are rich in sugar, unsaturated- polyunsaturated fatty acids, vitamins, polysaccharides, polyphenols and minerals (11). In particular, pomegranate seed oil contains high levels of phenolic compounds which is punicic acid, punicalagins (PNG), as well as important fatty acids such as linoleic acid, gallic acid and elagic acid (12, 13).

The high amount of punicic acid and PNG in pomegranate seed oil provides many beneficial biological effects such as anti-inflammatory, antioxidant, anti-apoptotic, anticancer and so on (14, 15). Other fatty acids found in the seed oil also have strong antioxidant and anti-inflammatory effects. Therefore, all these conditions suggest that the pomegranate seed oil may have serious therapeutic effects. Many experimental studies have shown pomegranate seed oil or PNGs can be used as natural drugs in the future. However, there is no study in the literature about pomegranate and its effects of ovarian ischemia and reperfusion injury. Therefore, we aimed to investigate the effects of pomegranate seed oil on experimental ovarian ischemia/reperfusion injury in rats.

## Materials and Methods

A total of 56 female albino Wistar rat were used in the experiments. Each rat weighed 200–250 g, and all were obtained from Ataturk University’s Experimental Animal Laboratory at the Medicinal and Experimental Application and Research Centre. The animal experiments and procedures were performed in accordance with national guidelines for the use and care of laboratory animals and approved by Kafkas University’s Local Animal Care Committee (28.01.2016-2016/19). The rat was housed in standard plastic cages on sawdust bedding in an air-conditioned room at 22±1 ^°^C. Standard rat food and tap water were given *ad libitum*.

***Chemicals***

All of the chemicals used in our laboratory experiments were purchased from Sigma Chemical Co (Munich, Germany). *Punica granatum* spp., known as Hicaz in our country, was purchased from city of Mersin (obtained year: 2016). Thiopental sodium was obtained from IE Ulagay AS (Istanbul, Turkey).

***Preparation of pomegranate seed oil extract***

Essential oils of the plants were obtained on a Clevenger (Wisd-Wise Therm) device by water vapor distillation. For this purpose, the fruits were dried and then the seeds were separated. One hundred and sixty g of the plant was pulverized in the shredder. The sample was placed in a glass balloon and 1600 ml of distilled water was added to it, then placed in a Clevenger apparatus and the apparatus was operated. After the evaporation started, it was left to stand for 3 hr. During this time, the hydrosol accumulated in the Clevengerin collection tube was taken in a sterile separate bottle. After the taken last hydrosol accumulated in the collection tube, the remaining volatile oil was stored in the dark bottles in the refrigerator at + 4 ^°^C until used in the experiment.

**Figure 1:**
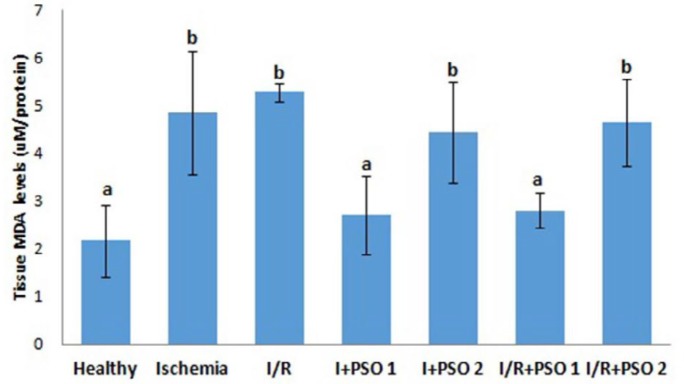
Effects of PSO on changes in the MDA level in ovarian ischemia and reperfusion injury. Means in the same column with the same letter are not significantly different; means in the same column with different letters indicate significant differences between the groups. *P*<0.05 (PSO: pomegranate seed oil, I/R: ischemia and reperfusion); MDA: Malondialdehyde

**Figure 2:**
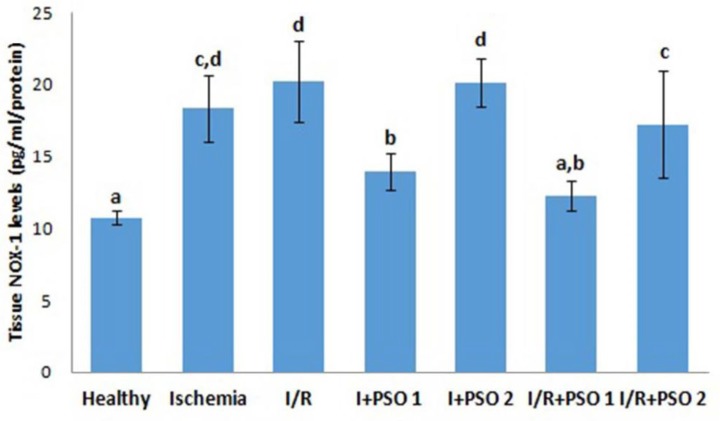
Effects of PSO on changes in the NOX1 level in ovarian ischemia and reperfusion injury. Means in the same column with the same letter are not significantly different; means in the same column with different letters indicate significant differences between the groups. *P*<0.05 (PSO: pomegranate seed oil, I/R: ischemia and reperfusion); NOX1: NADPH oxidase 1

**Figure 3:**
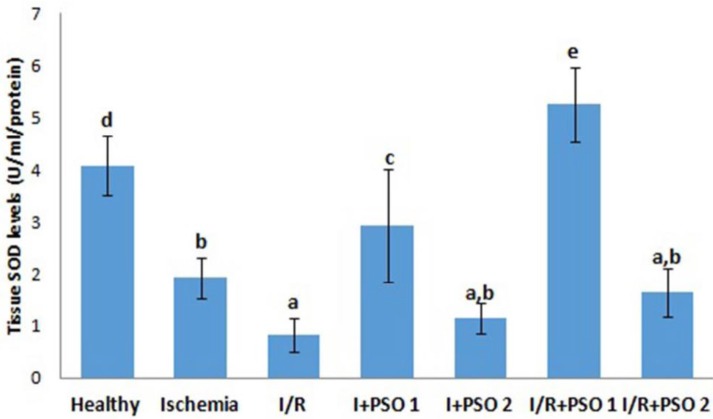
Effects of PSO on changes in the SOD activity in ovarian ischemia and reperfusion injury. Means in the same column with the same letter are not significantly different; means in the same column with different letters indicate significant differences between the groups. *P*<0.05 (PSO: pomegranate seed oil, I/R: ischemia and reperfusion); SOD: Superoxide dismutase

**Figure 4:**
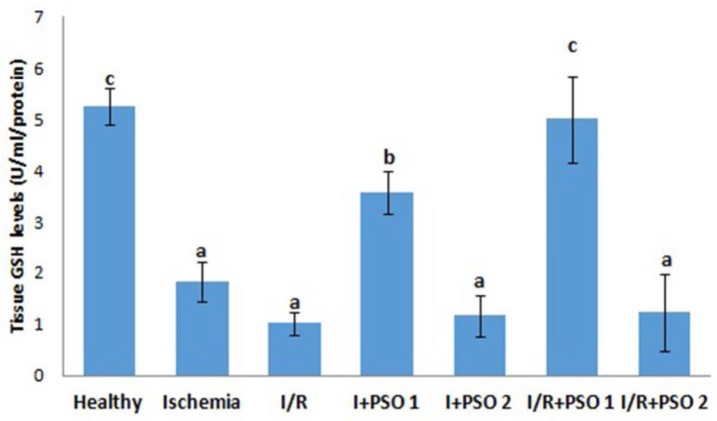
Effects of PSO on changes in the GSH levels in ovarian ischemia and reperfusion injury. Means in the same column with the same letter are not significantly different; means in the same column with different letters indicate significant differences between the groups.* P*<0.05 (PSO: pomegranate seed oil, I/R: ischemia and reperfusion); GSH: Glutathione

**Figure 5:**
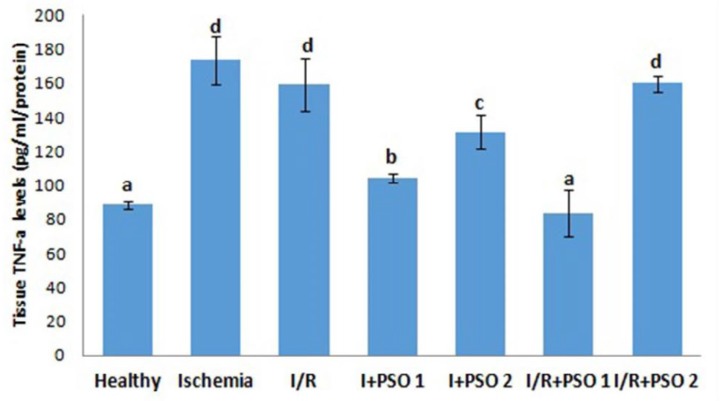
Effects of PSO on changes in the TNF-a levels in ovarian ischemia and reperfusion injury. Means in the same column with the same letter are not significantly different; means in the same column with different letters indicate significant differences between the groups. *P*<0.05 (PSO: pomegranate seed oil, I/R: ischemia and reperfusion)

**Figure 6:**
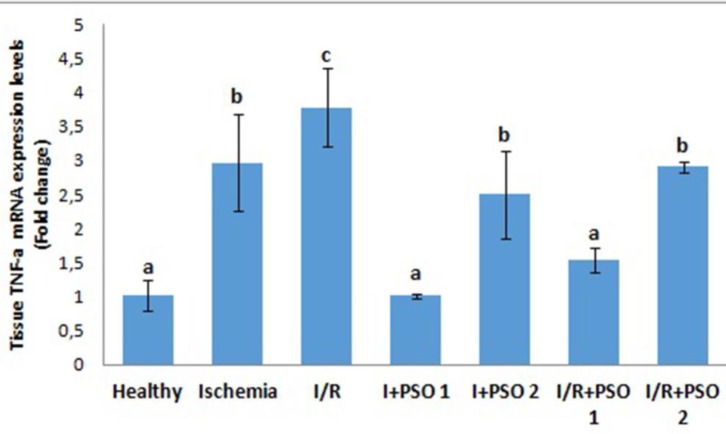
Effects of PSO on changes in the TNF-a mRNA expression in ovarian ischemia and reperfusion injury. Means in the same column with the same letter are not significantly different; means in the same column with different letters indicate significant differences between the groups.* P*<0.05 (PSO: pomegranate seed oil, I/R: ischemia and reperfusion)

**Figure 7:**
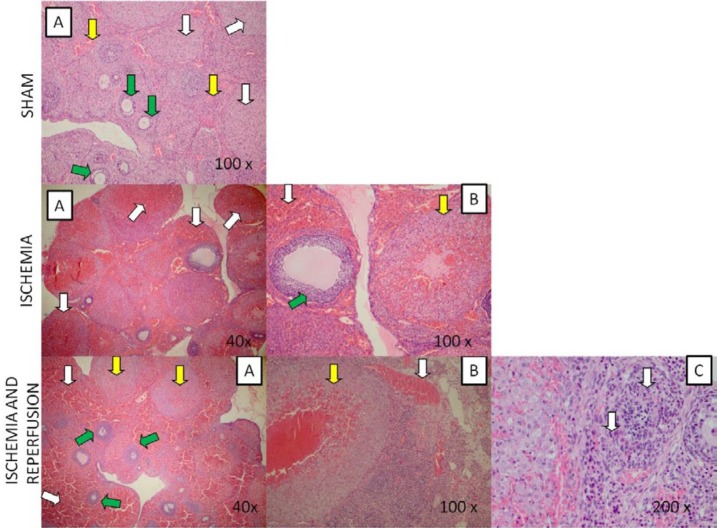
Sham: A) 100x, H-E staining results of Sham group. White arrow: corpus luteum, yellow: mild vascular congestion, green: ovarian follicles. Ischemia: A) 40x, H-E staining results of ischemia. White arrow: severe hemorrhage B) 100x, White arrow: severe hemorrhage, yellow: corpus luteum, green: ovarian follicles. Ischemia and Reperfusion: A) 40x, H-E staining results of I/R groups. White arrow: severe hemorrhage, yellow: corpus luteum, green: ovarian follicles. B) 100x, white arrow: vascular congestion, yellow: hemorrhagic corpus luteum C) 200x, H-E staining results of I/R groups. white arrow: apoptotic cells

**Figure 8:**
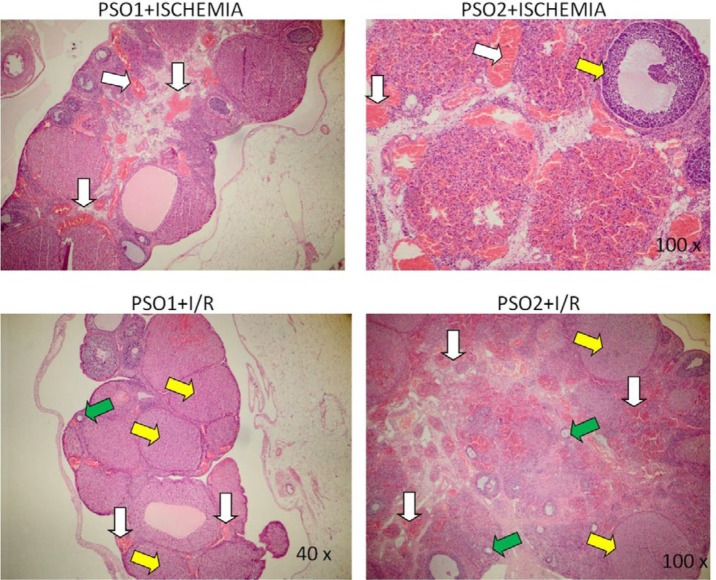
40x, H-E staining results of PSO1+ischemia groups. white arrow: vascular congestion. 100x, H-E staining results of PSO2+ischemia groups. White arrow: vascular congestion, yellow: ovarian follicle. 40x, H-E staining results of PSO1+I/R groups. white arrow: mild hemorrhagic and congestion, yellow: corpus luteum, green: primary ovarian follicle. 100x, H-E staining results of PSO2+I/R groups. moderate hemorrhagic and congestion, yellow: corpus luteum, green: primary ovarian follicle; PSO: Pomegranate seed oil

**Table 1:** Amounts of substance in 1.00-gram extract

Substance	Amounts (Relative, %)
Palmitic Acid (16:0)	0.9
Stearic Acid (18:0)	0.8
Oleic Acid (18:1)	2.1
Linoleic Acid (18:2)	2.6
Arachidic Acid (20:0)	0.3
Punicic Acid* (18:3)	45.1
Punicic Acid isomer (18:3)	14.8
Punicic Acid isomer (18:3)	14.8
Punicic Acid isomer (18:3)	15.0

% : flame ionization detector (FID)

* Punicic Acid: 9Z, 11E, 13Z octatrienoic acid

**Table 2:** Pathological scoring

Groups	Hemorrhage	Apoptotic cell	İncreased capillary permeability
Healthy	0	0	0
Ischemia	3	0	2
I/R	2	2	3
I+PSO1	1	0	1
I+PSO2	2	0	2
I/R+PSO1	1	0	1
I/R+PSO2	2,5	0	2

0: none, 1:mild , 2:moderate , 3:severe , I:ischemia

I/R: ischemia and reperfusion, PSO: pomegranate seed oil

***Analysis of ingredients in pomegranate seed oil extract by GC/MS***

Ingredients and fatty acids were detected in extract and quantitative determinations were made on the Agilent GC/MS (Germany).

The extract was dissolved in methanol to a concentration of 1 mg/ml and a stock solution was obtained and centrifuged at 10000 rpm for 5 min. Workup solutions were prepared by diluting the supernatant with phosphate buffer (pH = 2.5, 0.025 M). The working solutions were injected into the system after passing through the injection filter. Each injection was repeated three times (Table 1).

***Surgical technique and PSO administration***

Animals were anesthetized via intra-peritoneal (IP) injections of 25 mg/kg thiopental sodium. A longitudinal incision (2.5 cm) was created in the midline area of the lower abdomen. A small peritoneal incision was made, and the uterine horns and adnexa were located. Bilateral ovarian ischemia was induced by applying vascular clips below the ovaries. Three hours after ischemia, reperfusion was performed and then 3 hr after the rats’ ovaries were collected (16, 17). PSO was used in 0,32 and 0,64 ml/kg doses (IP injection). PSO administration to the treatment groups was performed 30 minutes before ischemia or reperfusion application as described below. 

Group 1, a sham operation, 

Group 2, ischemia for 3 hr

Group 3, ischemia (for 3 hr), reperfusion (for 3 hr after ischemia).

Groups 4 and 5, the rats were administered 0,32 and 0,64 ml/kg PSO. A half hour after the PSO administration, bilateral ovarian ISC was performed. Three hours after ischemia, the rats’ ovaries were collected. 

Groups 6 and 7, two and a half hours after the induction of ischemia, 0,32 or 0,64 ml/kg PSO was administered. A half hour after PSO administration reperfusion was performed. Three hours after reperfusion, the rats’ ovaries were collected.

Collected tissues were stored at -80 ˚C for biochemical and molecular analyses and at 10% formalin for histopathological analyses. 

***Biochemical analyses***

Rat tissues were kept at -86 ˚C. 100 mg of tissues from each rat was firstly perfused with PBS/heparin. All tissue samples from each rat were grinded in liquid nitrogen using a TissueLyser II grinding jar set (Qiagen, Hilden, Germany). Then they were centrifuged according to the manufacturer’s instructions. Subsequently, SOD activity, and MDA level, GSH level, NOX1 activity and TNF-α levels from each supernatant were measured in duplicate with highly sensitive kits (Cayman-706002, 10009055, 703002 (USA)), and Sunredbio-201611, 201-705 (china), respectively), specifically designed for rat tissue, according to the manufacturer’s instructions. The protein concentrations were determined by the Lowry method using commercial protein standards (Sigma Aldrich, Total protein kit-TP0300-1KT-(USA)). All the data was presented as the mean ± standard deviation (S.D.) results based on per mg of protein.

***Real-time PCR analyses***

 The samples for molecular analyses were immediately stored at -80 ^°^C. Before storage, all of the tissue samples from each rat were first perfused with PBS/heparin and then ground in liquid nitrogen with a TissueLyser II grinding Jar Set.

***RNA extraction and cDNA synthesis***

The ovarian tissues (20 mg) were stabilized in an RNA stabilization reagent (RNAlater, Qiagen) and then disrupted with the TissueLyser II (Qiagen) (2x2 minutes.). All of the RNA was purified using an RNeasy Mini Kit (Qiagen) and RNA samples were reverse-transcribed into complementary DNA with a high-capacity cDNA reverse transcription kit (Qiagen) according to the our previous study (18, 19). 

***Gene expression***

Relative rat TNF-α (PP-RA-300 taqman probe (Qiagen)) mRNA expression analyses were performed with StepOnePlus Real-Time PCR System technology (Applied Biosystems) using cDNA synthesized from RNA of ovary tissue described as previously (18, 19).

***Histopathological procedures***

The tissues reserved for histopathological examination were rapidly fixed in 10% buffered formalin for 24 hr. After fixation, each tissue sample was routinely processed described as previously (18, 19).

***Statistical analyses***

IBM SPSS statistical software Version 20.0 was used for the biochemical analysis. The results are presented as the means ± standard deviation (SD). Between-group comparisons for biochemical and molecular analyses were performed with one-way ANOVA and Duncan’s multiple comparison tests. Significance was accepted at *p*<0.05. Means in the same column with the same letter are not significantly different; means in the same column with different letters indicate significant differences between the groups according to the Duncan test.

## Results

***Oxidative stress marker***

*Tissue MDA levels*

In our study, MDA, an important marker of oxidative stress-related tissue damage, was measured by ELISA in all groups (Figure 1). MDA levels in ischemia and I/R groups were found to be significantly increased compared to healthy group (*P*=0.011, *P*=0.001, respectively). Reduced MDA levels observed in low dose treated PSO groups while no significant MDA level reduction observed in high dose PSO treated groups (*P*=0.023, *P*=0.861, respectively). It is thought here that PSO may have therapeutic benefits up to certain doses.

*Tissue NOX1 levels*

In our study, we assessed the level of NOX1, which has an important role in the formation of oxidative damage and whose expression has also been detected in oocyte cells (Figure 2). There was a significant increase in NOX1 level in the ischemia and I/R groups compared to the control group (*P*=0.044, *P*=0.001, respectively). It has been observed that PSO administration, especially in the 1st dose, prevented both ischemia and reperfusion injury and regresses the NOX1 levels (*P*=0.028, *P*=0.013, respectively). It is thought that increased NOX1 due to damage and decreased NOX1 due to treatment may have significant effects on the development of ovarian ischemia and reperfusion injury.

***Antioxidant markers***

*Tissue SOD activity and glutathione levels*

SOD and GSH are major defense mechanisms that protect our cells against oxidants. In situations such as ischemia, the reduction of antioxidant defense system in our tissues leads to the development and increases in damage. In our study, antioxidants decreased significantly in ischemia and I/R groups compared to healthy group (Figure 3-4) (for SOD; *P*=0.001, *P*=0.003, for GSH; *P*=0.015, *P*=0.001, respectively). PSO first dose application increased antioxidant defense systems in both ischemia and I/ R groups (for SOD; *P*=0.01, *P*=0.001, for GSH; *P*=0.015, *P*=0.037, respectively). However, the second dose of PSO did not show any protective effect on antioxidant defense system.

***Inflammatory markers***

*TNF-α tissue levels and mRNA expressions*

We assessed *TNF-α* level, a pro-inflammatory cytokine that contribute to the development of ischemia-reperfusion injury, both molecular and biochemically in our study (Figure 5-6). Tissue TNF-α expression increased 3-4 fold in ischemia and reperfusion groups compared to control (*P*=0.043, *P*=0.02, respectively). With PSO application, TNF-α expression was significantly down regulated, especially in the 1st dose (*P*=0.001). Biochemical findings of TNF-α are also parallel to TNF-α mRNA expression. While there was no significant difference between the ischemia and I/R groups of the tissue *TNF-α* level at the end of the ELISA measurement (*P*=0.318), TNF-α expression was more exaggerated in the I/R group than in the ischemia group (*P*=0.02). As it is determined here, increasing in the gene level of the inflammatory cytokines cannot be completely transformed into an active product.

***Pathological results***

Pathological examinations of ovary tissues were performed in H&E staining study (Table 2, Figure 7-8). Necrotic cells and neutrophil infiltration were not found in any group. In the ischemia and I/R groups, hemorrhagic foci were severe, but in the PSO group, especially in the first dose, the pathological changes were mild. While the capillary permeability increased in the ischemia and reperfusion groups, PSO application revealed to decrease capillary permeability. Moderate

## Discussion

We have shown the potential therapeutic effects of pomegranate oil in experimental ovarian I/R injury in rats via biochemical, molecular and pathological analyses.

Pomegranate is one of the most consumed fruit since ancient times for strong antioxidant and anti-inflammatory activity. Many pharmacological effects of fruit juice, peel and seed extracts of the pomegranate have been shown. In particular, pomegranate seed oil (PSO) is enriched punicic acids and punicalagins. It also contains many fatty acids (especially linoleic acid, gallic acid and elagic acid). Punicic acid, which is abundant in pomegranate seed, has common pharmacological properties. Previous studies demonstrated the protective effect of PSO in different experimental I/R models and PSO showed these effects by reducing oxidative stress, increasing antioxidants and preventing- limiting inflammation (20, 21).

Oxidative stress is the primary cause of ovarian ischemia and reperfusion damage. Disruptions of energy metabolism in the cells undergo stress, oxidant agents are started to be produced (20-22). MDA is the final degradation product of membrane proteins during oxidative stress. In experimental studies, MDA, an indicator of oxidative damage, has measured as a gold standard. In our study, oxidative stress significantly increased in ischemia and I/R groups, while PSO administration significantly reduced the MDA level. Previous studies demonstrated that PSO administration significantly decreased oxidative stress and MDA levels (23). In this respect, PSO exerted antioxidant effect against ovarian injury during ischemia and I/R.

During ischemia and reperfusion changing in the structure and function of many enzymes also contributes to aggravation of oxidative stress and inflammation. The most important of these are xanthine oxidase (XO) and NADPH oxidase (NOX) (24, 25). XO cause damage in acute phase of ischemia and reperfusion injury. NOX is involved in the late phase of I/R (3). In our study, 3-hr ischemia and 3-hr reperfusion model (a total 6-hr I/R model) were established. In the late phase of reperfusion, NOX increase oxidative damage and stimulates pro-inflammatory cytokines such as TNF-α. NOX is normally synthesized from phagocytic cells (26). Recently, it has been shown NOX expressed and synthesized in many tissues and cells and its subtypes 1 to 5. NOX1 has been shown to be present in ovaries and oocytes in experimental animals (27). NOX-2 is mainly derived from phagocytic cells (26). Unlike NOX2, NOX1 directly stimulates TNF-α expression (28). This suggests that NOX1 also may have important effects on acute inflammatory response in I/R injury. NOX1 also induces apoptosis of cells during I/R (29).

In our study, NOX1 level was found to be at a high level in the ischemia and I/R group and parallel to this, cells were shown to apoptosis after reperfusion. TNF-α level significantly increased in parallel with the level of NOX1 (28). TNF-α has an important role of developing inflammation and triggering apoptosis of cells. Ferreira *et al.* demonstrated that PSO has a strong anti-inflammatory and anti-nociceptive activity and it is proposed PSO may be used as alternatives for NSAIDs (30, 31). In another study, PSO and punicic acid exert anti-inflammatory effects on colon inflammation in rats. Current study, PSO and punicic acid inhibited TNF-α induced priming of NADPH oxidase by targeting the p38MAPKinase/Ser345-p47phox-axis (32). In this respect, current study demonstrated that the increased expression of TNF-α parallel with NOX1 caused both inflammation and the onset of apoptosis in the late phase of reperfusion. First dose of PSO administration significantly prevented irreversible damage related to improved NOX1 and TNF-α level.

There are many mechanisms in our body to prevent I/R injury. Antioxidants are responsible for the scavenging of the oxidants. SOD is known one of the most important antioxidant and GSH, another important antioxidant, allows free radicals and toxins to be removed by glucronyl conjugation. SOD and GSH protect our cells against the cytotoxic effects of free radicals. A reduction in SOD activity and GSH levels in ischemia and reperfusion may be due to the overproduction of superoxide radical anions (33-35).

In our study, PSO application significantly improved antioxidant activity in ischemia and I/R groups. However, PSO has shown very low pharmacological activity at high doses. This suggests that natural products may have therapeutic efficacy in certain dose ranges, it is clear that excessively consumed pomegranate will not have a therapeutic benefit.

We finally assessed the pathology of over-tissues to support all of these findings. Severe hemorrhage and increased capillary permeability were seen in the ischemia and reperfusion groups. Increased capillary permeability lead to exacerbation of inflammation during ischemia. This supporting multiple mechanisms have role in ischemia and reperfusion injury. It was also seen that in I/R group, damage progressed and apoptosis occurred. In previously, It is demonstrated that anti-apoptotic effect of PSO on brain hypoxic ischemia through inhibition of caspase 3 (36, 37). In our study, PSO administration has been able to protect the cells especially in the first dose by showing anti-apoptotic effect. If there is no operation and medical treatment during I/R, the damage is irreversible and the person will be sterile. 

## Conclusion

PSO has shown protective effect on ovarian ischemia and reperfusion injury by decreasing oxidative stress, improving TNF-α and NOX1 levels, increasing antioxidant defense system and preventing development of apoptosis. It has been shown in our study that PSO may be a potential therapeutic agent in the ovarian-I/R injury. However, our study should be supported by more detailed experimental studies and clinical trials. Also, the effects of punicalagins and other ingredients of PSO should be demonstrated directly on ovarian I/R injury.
